# Naturalizing psychopathology—towards a quantitative real-world psychiatry

**DOI:** 10.1038/s41380-021-01322-8

**Published:** 2021-10-20

**Authors:** Juha M. Lahnakoski, Simon B. Eickhoff, Juergen Dukart, Leonhard Schilbach

**Affiliations:** 1 Institute of Neuroscience and Medicine, Brain & Behaviour (INM-7), Research Centre Jülich, Jülich, Germany; 2 Institute of Systems Neuroscience, Medical Faculty, Heinrich Heine University Düsseldorf, Düsseldorf, Germany; 3 LVR-Klinikum Düsseldorf, Düsseldorf, Germany; 4 Medical Faculty, Ludwig-Maximilians-Universität, München, Germany

**Keywords:** Predictive markers, Neuroscience, Physiology, Psychology

Psychiatric disorders continue to be on the rise around the globe. Meanwhile, efforts and investments directed to early diagnosis and appropriate interventions for mental health problems are lagging resulting in ‘substantial loss of human capabilities and avoidable suffering’ [1]. A major component of our inability to address mental health problems resides in the persistent lack of objective measures for evaluating such difficulties in the daily life of individuals, complicating the detection of clinically relevant changes in the patients’ well-being [2]. Similarly, most studies into the neurobiology of psychiatric disorders lack a detailed description of individual functioning despite influential calls for quantitative approaches to psychopathology.

Psychiatric conditions are often particularly reflected through, and exacerbated by, difficulties in social functioning in everyday life [3]. Yet, current diagnostic evaluations primarily take the form of limited interactions in artificial clinical settings. While these types of diagnostic procedures have clear clinical value, they are often subjective and qualitative in nature. Reports provided by the patient may suffer from memory biases and can also depend on the rapport and trust between the patient and the clinician. Concurrently, standardized questionnaires probe a limited set of functional impairments and are administered only infrequently during clinical consultations. With the slow onset and the non-specific and transient nature of functional impairments in psychiatric conditions, these limitations may prevent a more comprehensive description and early detection of psychopathology [1].

**Fig. 1:**
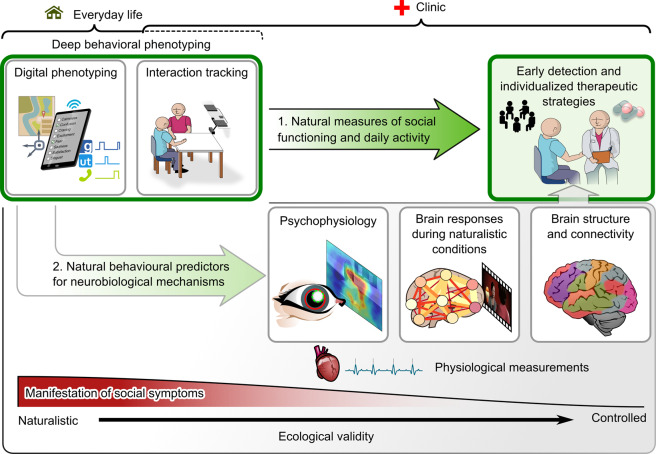
Graphical summary of a framework for multiscale assessment of psychopathology. Our framework emphasizes deep behavioural phenotyping in natural conditions of everyday life and minimally constrained interactions (green box, top left). These approaches can (1) provide objective measures of daily functioning that can directly guide early detection and individual intervention strategies in the clinic, thus helping with the limitations of in-clinic evaluations. Additionally, (2), the individual measures of daily functioning may help to uncover neurobiological markers of the symptom dimensions without relying on heterogeneous disease labels. These findings may then feed back to clinical practice.

We argue that a thorough understanding of the real-world manifestations and implications of psychiatric disorders is the key for the field to move towards the development of more effective personalized assessments and interventions. To address this, we outline a general multilevel framework for deep behavioural phenotyping to guide the scientific inquiry into the behavioural and neural mechanisms of psychopathology and to delineate specific therapeutic interventions (Fig. 1). This approach emphasises an objective and continuous evaluation of psychopathology in everyday life and naturalistic social interactions (green box, top left) to guide clinical practice and scientific research.

The most debilitating symptoms of psychiatric disorders may only manifest in everyday life, where patients must cope with multiple aspects of life simultaneously (illustrated by the red scale at the bottom of Fig. 1). Current technologies can enable detailed and objective monitoring of the impact of mental health problems on everyday life of the patient based on their natural behavioural patterns. One way to achieve this is by measuring daily behaviour using personal smart devices, often referred to as “digital phenotyping” [4]. Using these devices, everyday life behaviour can be assessed passively, for example by objectively measuring the amount of physical activity or phone-based social interaction. Moreover, temporal patterns of user interface inputs may provide valuable insights into psychomotor symptoms such as agitation or retardation.

Importantly, these passive measures of daily behaviour can be complemented by subjective ecological momentary assessments providing real-time self-reported measures of patients’ well-being. Such subjective momentary evaluations may prove crucial to understanding changes in passively monitored behavioural patterns. For example, while single passive measures may not be sufficiently discriminative, negative mood evaluations in combination with reductions in locomotive activity, social application usage and speed of typing may represent early signs of a depressive episode. Detecting such patterns offers a chance for early intervention to avoid hospitalization (arrow 1. In Fig. 1).

While such digital phenotyping can address macroscopic aspects of social and motor functioning, it lacks specificity for assessing how these problems relate to difficulties the patients face during real-life social interactions. To address this, recent studies have started to objectively measure behaviour during social interactions using motion tracking techniques to detect e.g. individual differences related to interaction success [5] and behavioural predictors for psychiatric disorders and therapeutic outcomes [6]. Such tracking techniques can be used to evaluate psychomotor symptoms in more detail based on face and whole-body movements. Moreover, some of the symptoms or behavioural tendencies of a person may manifest more strongly during a dyadic real-time interaction rather than during interaction with a digital user interface. In a clinical setting, such fine-grained characterization of behaviour during dyadic interactions may assist a clinician to reveal subtle early signs for interpersonal difficulties before symptoms develop into a fully-fledged psychiatric disorder. Such behavioural measures can also be extremely beneficial for elucidating the neurobiological underpinnings of psychiatric disorders and the behavioural and neural mechanisms of psychotherapeutic interventions (arrow 2. In Fig. 1). Moreover, combining behavioural and physiological measurements during immersive virtual reality may expand the behaviours that can be evaluated, and have shown promise as generalizable predictors of individual susceptibility to stress, which is an important contributing factor to psychiatric disorders [7].

To date, most studies focusing on the neural bases of psychiatric disorders performed categorical comparisons of psychiatric disorders while overlooking the extensive variability in how the disorders manifest in individual patients. Whilst replicable brain-based group differences appear to exist [8], group-level effect sizes are often small and the measures may be more indicative of the general level of psychopathology across multiple disorders [9]. Progress in machine learning techniques may improve the specificity of imaging-based biomarkers in the future. However, it is also increasingly recognized that neurobiological changes might be more strongly associated with combinations of dimensions of psychopathology than general clinical labels. Comprehensive behavioural phenotyping may help uncover clinically relevant behavioural patterns and provide a window into individual, rather than group-based, neurobiological underpinnings of psychiatric disorders.

Several approaches could be used to measure the neurobiological and physiological correlates of psychiatric disorders from stable anatomical properties to short-term functional changes during naturalistic experimental conditions (we illustrate some options in Fig. 1). Behavioural symptoms are likely not equally reflected at all levels of this continuum. For example, transient and context-specific difficulties in social life may not be reflected as anatomical differences at scales visible in standard anatomical MRI images. By contrast, exposure to disorder-relevant stimuli may elicit readily detectable activity differences. Conversely, these differences may reflect the effect of the conditions while the causes may lie in pathophysiological alterations at a different scale. Thus, it is important to critically evaluate which measures are most reflective of, and contributing to, psychopathology.

Such insights from combining behavioural and neuroimaging markers of psychopathology may be key for detecting clinically applicable biomarkers for psychiatric disorders that can feed back into clinical practice (grey arrow in Fig. 1) and enable novel insights into the brain mechanisms underlying individual psychopathology [10].
